# PT symmetry protected non-Hermitian topological systems

**DOI:** 10.1038/s41598-018-35795-5

**Published:** 2018-11-27

**Authors:** C. Yuce, Z. Oztas

**Affiliations:** Department of Physics, Eskisehir Technical University, Eskisehir, 26555 Turkey

## Abstract

We study PT symmetry protected topological phase in non-Hermitian 1D and 2D systems. We show that topological phase exist in non-Hermitian PT symmetric systems for both gapped and gapless systems and discuss appearance of exceptional points. We apply our formalism to a complex extension of the SSH model, topological semimetals and nodal superconductors.

## Introduction

Topological phase in non-Hermitian systems is an emergent field of study that has applications in topological photonics^1^. Although topological phase in Hermitian systems have been well understood, little is known about its generalization to non-Hermitian systems. It is generally believed that non-Hermitian systems present new topological physics inaccessible in Hermitian systems. However, the existence of topological phase in non-Hermitian system was controversial for a long time^2–5^. It was concluded that topological phase is not stable in non-Hermitian systems since the energy eigenvalues are not real. Fortunately, a few years ago, stable topological phase was shown to exist in a non-Hermitian ${\mathscr{P}}{\mathscr{T}}$ symmetric system, where ${\mathscr{P}}$ and ${\mathscr{T}}$ are parity and time reversal operators, respectively^6,7^. In^6^, it was theoretically predicted that stable topological phase is compatible in a non-Hermitian Aubry-Andre model^6^. A topological zero energy state was observed through fluorescence microscopy in a lattice of waveguides with staggered hopping amplitudes^7^. Since then, topological photonics with gain and loss have attracted great deal of attention. In this sub-field, mainly one dimensional problems have been studied in the literature^8–14^. Of special interest is the one dimensional complex extension of the Su-Schrieffer-Heeger (SSH) model, which has topological zero energy modes^15,16^. In the paper^17^, it was theoretically shown that chiral topological edge modes can be realized in honeycomb lattices of ring resonators with asymmetric gain-loss couplings. It was also discussed in that paper that exceptional points of the bulk Hamiltonians and the topological edge modes are related. Not only topological insulators but also topological superconductors was generalized to non-Hermitian systems. Majarona modes in topological superconductors have been studied in some non-Hermitian systems^18–22^. Floquet topological insulators^23^ that appear in time-periodic systems was studied in systems with gain and loss^24^.

Despite the progress of non-Hermitian topological photonics, the topic is still in its infancy. There is no general framework to understand topological phase in the presence of gain and loss. There are still many issues that has not been understood fully such as bulk-boundary correspondence^25^, topological invariants^26^ and classification of topological systems with symmetries^27,28^ in non-Hermitian systems. Two topologically distinct gapped systems in the same symmetry class can be continuously deformed each other without closing the band gap. Note that topological phase is not restricted to gapped systems. Semimetals and nodal superconductors has also nontrivial band topology. Their bulk gap closes at certain points in the Brillouin zone. These nodal systems can be protected by both nonspatial symmetries and spatial lattice symmetries^29^. In this paper, we are interested mainly in the combined parity-time symmetry for two-band models. We study parity-time symmetric non-Hermitian topological phase. We apply our formalism to gapped and gapless non-Hermitian systems in 1*D* and 2*D*.

## Parity-Time Symmetric Systems

Symmetries play important roles in the classification of topological insulators and superconductors. To study symmetry-protected topological phase in non-Hermitian systems, we begin with definitions of four basic types of symmetries for translational invariant systems

1 $$\begin{array}{c}{\mathscr{T}}\, {\mathcal H} (-\,{\bf{k}}){{\mathscr{T}}}^{-1}= {\mathcal H} ({\bf{k}});\,\,{ {\mathcal H} }^{2}=\mp \,1\\ {\mathscr{C}}\, {\mathcal H} (-\,{\bf{k}}){{\mathscr{C}}}^{-1}=-\, {\mathcal H} ({\bf{k}});\,\,{{\mathscr{C}}}^{2}=\mp \,1\\ {\mathscr{S}}\, {\mathcal H} ({\bf{k}})\,{{\mathscr{S}}}^{-1}=-\, {\mathcal H} ({\bf{k}});\,\,{{\mathscr{S}}}^{2}=1\\ {\mathscr{P}}\, {\mathcal H} ({\bf{k}}^{\prime} )\,{{\mathscr{P}}}^{-1}= {\mathcal H} ({\bf{k}});\,\,{{\mathscr{P}}}^{2}=1\end{array}$$

where *k′* indicates which component of the quasi-momentum is inverted. Here ${\mathscr{T}}$ and ${\mathscr{C}}$ are the antiunitary time-reversal and particle-hole operators, and ${\mathscr{S}}$ and ${\mathscr{P}}$ are the unitary chiral and parity operators, respectively. Note that the chiral operator can also defined as ${\mathscr{S}}={\mathscr{T}}{\mathscr{C}}$. Here, we restrict ourself to 1*D* and 2*D* systems. In one dimension, there is unique definition of the parity operator: *k′* = −*k*. However in 2*D*, the parity operator inverts either one or both components of quasi-momentum, *k′* = ($\mp$*k*_*x*_, ±*k*_*y*_) or **k**′ = (−*k*_*x*_, −*k*_*y*_).

To study ${\mathscr{P}}{\mathscr{T}}$ symmetry protected topological phase in non-Hermitian systems, we consider the following topologically insulating two band model

2 $$ {\mathcal H} (k)={d}_{x}({\bf{k}})\,{\sigma }_{x}+{d}_{y}({\bf{k}})\,{\sigma }_{y}+i\,g\,{d}_{z}({\bf{k}})\,{\sigma }_{z}$$

where *σ*_*x*_, *σ*_*y*_ and *σ*_*z*_ are Pauli matrices, *g* is the non-Hermitian degree and **k** is the crystal momentum defined in the first Brillouin zone.

One can easily see that this non-Hermitian Hamiltonian and its Hermitian part (*g* = 0) are both ${\mathscr{P}}{\mathscr{T}}$ symmetric: ${\mathscr{P}}{\mathscr{T}}={\sigma }_{x}K$ with ${({\mathscr{P}}{\mathscr{T}})}^{2}=1$, where *K* is the complex conjugation operator. Therefore, we see that the term with *g* can safely be added to the Hermitian Hamiltonian to study ${\mathscr{P}}{\mathscr{T}}$ symmetric topological phase. As opposed to the Hermitian limit, ${\mathscr{P}}{\mathscr{T}}$ symmetry of the non-Hermitan system may be spontaneously broken, i. e., the eigenstates of^2^ are no longer the eigenstates of the ${\mathscr{P}}{\mathscr{T}}$ operator. In this case, exceptional points occurs and then the system enters into region where complex energy eigenvalues appear. A question arises. Can we adiabatically deform the non-Hermitian system *g* ≠ 0 into the Hermitian system *g* = 0 without closing the bulk gap and breaking the discrete symmetries? If the answer is Yes, then the Hermitian and non-Hermitian Hamiltonians are said to be topologically equivalent under the same symmetry group. To answer this question, let us give the energy eigenvalues of (2): ${E}_{\mp }=\mp \,\sqrt{{d}_{x}^{2}+{d}_{y}^{2}-{g}^{2}{d}_{z}^{2}}$. The band gap closing occurs at some particular values **k**_0_ in the Hermitian limit, *d*_*x*_(**k**_0_) = *d*_*y*_(**k**_0_) = 0. Assume that *d*_*z*_(*k*) are equal to zero at these particular values of the crystal momentum, *d*_*z*_(**k**_0_) = 0. Then non-Hermitian part of the Hamiltonian plays no role on band gap closing and opening points in *k*-space. As long as the ${\mathscr{P}}{\mathscr{T}}$ symmetry is not spontaneously broken, complex energy eigenvalues don’t appear in the system during topological phase transition and the Hermitian and non-Hermitian systems are topologically equivalent. To illustrate our idea, let us now give some examples. Consider the following complex extension of the SSH model, which is a one dimensional tight-binding model with alternating hopping amplitudes

3 $$ {\mathcal H} (k)=(\nu +\omega \,\cos \,k){\sigma }_{x}+\omega \,\sin \,k\,{\sigma }_{y}+ig\,{d}_{z}(k)\,{\sigma }_{z}$$

where *ν* > 0 and *ω* > 0 are hopping amplitudes. We analyze this non-Hermitan Hamiltonian for two different choices of *d*_*z*_(*k*): *d*_*z*_ = 1 and *d*_*z*_ = sin*k*, where the latter one satisfies the above condition while the former one does not. This Hamiltonian has particle-hole symmetry with ${\mathscr{C}}={\sigma }_{z}K$ for *d*_*z*_ = 1 and time reversal symmetry with ${\mathscr{T}}=K$ for *d*_*z*_ = sin*k*. Both cases are ${\mathscr{P}}{\mathscr{T}}$ symmetric.

Let us now study energy eigenvalues and topological phase transition in our system for each *d*_*z*_. The energy eigenvalues for (3) are given by ${E}_{\mp }=\mp \,\sqrt{{\nu }^{2}+{\omega }^{2}+2wv\,\cos (k)-{g}^{2}{d}_{z}^{2}}$. Without loss of generality, we suppose *ν* is constant and *ω* is varied to see topological phase transition. Consider first the Hermitian limit, *g* = 0, in which two bands are symmetrically arranged about zero energy and separated by a gap of |*ω* − *ν*|. If we deform the Hamiltonian by varying *ω* from a value *ω* > *ν* to a value *ω* < *ν* for fixed *ν*, we see that the band gap closes and reopens at *ω* = *ν*. This shows us that topological phase transition occurs exactly at *ω* = *ν* and the cases with *ω* > *ν* and *ω* < *ν* are topologically distinct. As opposed to the Hermitian systems, the band gap doesn’t reopen just after closing in the non-Hermitian case as we see below.

(*i*) *d*_*z*_(*k*) = 1: In this case, the spectrum is real as long as the system is gapped. The non-Hermitian degree decreases the band gap and hence the band gap vanishes at a bigger value of *ω*. The band gap closing at *ω* = *ν* + *g* leads to the appearance of exceptional points at *k* = $\mp$*π*. Decreasing *ω* below than this value doesn’t reopen the band gap. Instead complex energy eigenvalues appear in the system. As a result, ${\mathscr{P}}{\mathscr{T}}$ symmetry is spontaneously broken during the topological phase transition. This happens in the interval *ν* − *g* < *ω* < *ν* + *g*. Fortunately, the band becomes gapped and the ${\mathscr{P}}{\mathscr{T}}$ symmetry is restored again in the region *ω* < *ν* − *g*. Therefore, the two systems with *ω* > *ν* + *g* and *ω* < *ν* − *g* are topologically distinct. A question arises. Can we find topological edge states with real valued energy eigenvalues at the interface of these two topologically distinct systems with real spectra? As opposed to Hermitian systems, our non-Hermitian system breaks ${\mathscr{P}}{\mathscr{T}}$ symmetry during topological phase transition. So, topological edge states are not generally stable since they have complex energy eigenvalues^30^. To overcome this problem, some methods have been introduced in the literature^6,7^. To this end, we stress that the Zak phase is independent of the non-Hermitian strength *g* and is equal to zero (a constant) when *ν* > *ω* (*ν* < *ω*). Note that the Zak phase is defined as $\gamma ={\int }_{-\pi }^{\pi } < \alpha |i{\partial }_{k}\alpha  > dk$, where |*α* >  is the eigenstate for the lower energy eigenvalue.

(*ii*) *d*_*z*_(*k*) = sin*k*: There are three different regions for this choice. *1-)* |*g*| < *ν; 2-) g* = *ν; 3-)* |*g*| > *ν*. Consider the first region, |*g*| < *ν*. In this case, the band gap does not depend on the non-Hermitian strength *g*. As it is in the Hermitian SSH system, the band gap closes and reopens at *ω* = *ν*, which shows that *ω* = *ν* is the topological phase transition point. We emphasize that exceptional points don’t appear even if the two bands touches at *k* = $\mp$*π* when *ω* = *ν*. (eigenvectors don’t coalesce). Therefore, ${\mathscr{P}}{\mathscr{T}}$ symmetry is not broken and the system admits real valued band structure. As a result, we see that topological phase transition occurs without passing through exceptional points. Consider now the second region with *g* = *ν*. If *ω* = *ν*, band gap closes at *k* = $\mp$*π*. In this case, band gap closing points are exceptional points since the eigenstates coalesce. We stress that the exceptional points don’t disappear in the system even if we reduce *ω*. Instead, their positions change. More specifically, exceptional points shift in the *k*-axis according to *cos*(*k*) = −*ω*/*ν*. They are at *k* = $\mp$*π* when *ω* = *ν* and approach each other as *ω* is decreased. Note that they would meet at *k* = 0 if *ω* takes a negative value. At *ω* = −*ν*, they annihilate each other and band is gapped out. For positive values of *ω* and *ν*, exceptional points can appear but the spectrum is always real valued. Since the band gap doesn’t reopen, no topological phase transition occurs in this case. Finally, consider the third region, *g* > *ν*. In this case, the system is gapped if *ω* > *g* and exceptional points occur when *ω* = *g*. The two exceptional points move towards each other in *k*-axis as *ω* = *g* are increased at fixed *ν*. If we reduce *ω* below than the non-Hermitian strength, *ω* < *g* then the ${\mathscr{P}}{\mathscr{T}}$ symmetry is spontaneously broken and complex energy eigenvalues appear. Having studied each cases separately, we say that the non-Hermitian Hamiltonian (3) can be deformed into its Hermitian part without breaking ${\mathscr{P}}{\mathscr{T}}$ symmetry or closing the gap provided *g* < *ν* if *d*_*z*_ = sin *k*. In other words, Hermitian and non-Hermitian systems have the same topology. To this end, we give the lattice form of the Hamiltonian^3^ for *d*_*z*_ = sin *k*. It can be found using $$H={\sum }_{k=0}^{\infty }\,({c}_{A,k}^{\dagger },{c}_{B,k}^{\dagger }) {\mathcal H} (\begin{array}{c}{c}_{A,k}\\ {c}_{B,k}\end{array})$$ and a Fourier transformation ${c}_{i,k}=1/\sqrt{N}\,{\sum }_{k}\,{e}^{ikn}{c}_{i,n}$, where *c*_*i*,*n*_ and ${c}_{i,n}^{\dagger }$ are the annihilation and creation operators localized at site *n* for the sub-lattice *i*, respectively and *N* is the total number of lattice sites. Therefore the corresponding lattice form of the Hamiltonian with *d*_*z*_(*k*) = sin *k* is given by $H={\sum }_{n=1}\,\nu \,{c}_{A,n}^{\dagger }{c}_{B,n}+\omega \,{c}_{A,n+1}^{\dagger }{c}_{B,n}+h\mathrm{.}c\mathrm{.}+\frac{g}{2}({\sum }_{n}\,{c}_{A,n}^{\dagger }{c}_{A,n+1}-{c}_{B,n}^{\dagger }{c}_{B,n+1}-h\mathrm{.}c)$, where h.c. means Hermitian conjugate. The first part of the Hamiltonian is the well known SSH Hamiltonian while the other term with *g* is next-nearest-neighbor (NNN) hopping term. The asymmetric character of NNN hopping term makes the systems non-Hermitian.

Having studied a 1*D* problem, let us explore topological phase in 2*D* ${\mathscr{P}}{\mathscr{T}}$ symmetric insulators. Unfortunately, we have not found 2*D* gapped ${\mathscr{P}}{\mathscr{T}}$ symmetric topological insulator with real spectrum. Let us now extend our idea to gapless 2*D* topological systems. In 2*D*, there are multiple Dirac points on *k*_*x*_, *k*_*y*_ planes. Therefore, exceptional points occur at multiple points in a ${\mathscr{P}}{\mathscr{T}}$ symmetric non-Hermitian systems. Below we study some examples.

Consider a two-band Hamiltonian with two atoms per unit cell in 2*D*. Therefore the Hamiltonian is given in terms of the Pauli spin matrices. Suppose that alternating gain and loss are added into the system. Around the Dirac points, which come in pairs, the lowest order Hamiltonian is given by^31^

4 $$ {\mathcal H} (k)=({\rm{\Delta }}+\frac{{k}_{x}^{2}}{2m}){\sigma }_{x}+v{k}_{y}{\sigma }_{y}+ig{\sigma }_{z}$$

where Δ is a real valued constant, *m* is the effective mass and *v* has the dimension of velocity. As can be seen, this Hamiltonian is ${\mathscr{P}}{\mathscr{T}}$ symmetric, where ${\mathscr{P}}{\mathscr{T}}={\sigma }_{x}K$. The resulting energy eigenvalues read ${E}_{\mp }\,=$ $\mp \,\sqrt{{({\rm{\Delta }}+{k}_{x}^{2}/2m)}^{2}+{v}^{2}{k}_{y}^{2}-{g}^{2}}$, which is linear in one direction and quadratic in the other. In the absence of gain and loss, *g* = 0, the system becomes topologically trivial when Δ > 0 and topological phase transition occurs and the system has semi-metallic phase at Δ = 0. The presence of gain and loss shifts the value of Δ for the semi-metallic phase to Δ = *g*. As opposed to its Hermitian counterpart, the Dirac point is an exceptional Dirac point and the states at the tips of Dirac cones coalesce. If |Δ| < *g*, then states with complex energy eigenvalues appear around the Dirac point. In this case, exceptional points don’t occur at a single point, *k*_*x*_ = *k*_*y*_ = 0, but instead on a ring centered at *k*_*x*_ = *k*_*y*_ = 0. More precisely, the set of exceptional points forms an exceptional ring and complex energy eigenvalues appear inside the exceptional ring. Although the lowest order Hamiltonian is good enough to understand the basic idea, let us study a full lattice model where the motion and merging of Dirac points may be realized. Consider the graphene structure with one of the three hopping parameters between nearest neighbors different from the two others. This *strained graphene* is not experimentally feasible in carbon-based graphene^31^ but it was realized experimentally in photonics^32^. The corresponding complex Hamiltonian has the form of (2) with

5 $$\begin{array}{rcl}{d}_{x} & = & {c}_{1}\,\cos ({k}_{x})\,+\,2\,{c}_{2}\,\cos (\frac{3}{2}{k}_{x})\,\cos (\frac{\sqrt{3}}{2}{k}_{y}),\\ {d}_{y} & = & {c}_{1}\,\sin ({k}_{x})\\  &  & +\,2\,{c}_{2}\,\sin (\frac{3}{2}{k}_{x})\,\cos (\frac{\sqrt{3}}{2}{k}_{y})\end{array}$$

where *c*_1_ and *c*_2_ are real valued parameters. For the regular graphene, the hopping parameters are given by *c*_1_ = *c*_2_ = 1. The uncompressed lattice exhibits the six Dirac points arranged in a regular hexagon. Each two of the Dirac points have opposite Berry phase and they are topologically protected. The Dirac points approach each other by tuning *c*_1_ and they meet in such a way that total Berry phases vanishes at *c*_1_ = 2*c*_2_. The system is gapped and topologically trivial if *c*_1_ > 2*c*_2_. In the experiment^32^, generation of edge states on the zigzag edge and destruction of them on the bearded edge were observed. Suppose now that *d*_*z*_ = 1. Complex energy eigenvalues appear in a small interval around these Dirac points. The upper and lower bands touch each other at some *k*_*x*_, *k*_*y*_ points other than the Dirac points of the regular graphene. These points form exceptional rings which are arranged in a regular hexagon when *c*_1_ = *c*_2_ = 1. Note that they are not intersected if the non-Hermitian degree is not sufficiently large. These exceptional rings shrink and approach each other in pairs as *c*_1_ is increased for fixed *c*_2_. At a critical value of *c*_1_, they merge and these exceptional rings disappear. If *c*_1_ is increased above than this critical value, the system is gapped and the band becomes real valued. Note that the ${\mathscr{P}}{\mathscr{T}}$ symmetry is spontaneously broken in the semi-metallic phase. Finally, we say that finding *d*_*z*_(**k**) for which the ${\mathscr{P}}{\mathscr{T}}$ symmetry is not broken in 2*D* is experimentally impractical. Such a *d*_*z*_(**k**) must vanish at each Dirac points and it should not lead to complex energy eigenvalues around the Dirac points.

Let us give another example in 2*D*. We apply our formalism to topological superconductors (TSC). Here we are not interested in fully gapped TSC but in nodal (zero-gap) superconductor. We start with the model introduced in^29^ and add a complex term as follows

6 $$ {\mathcal H} (k)=ig{d}_{x}{\sigma }_{x}+\,\sin \,{k}_{x}{\sigma }_{y}+(\lambda \mp \,\cos \,{k}_{y})\,{\sigma }_{z}$$

where *λ* is a real valued constant. Note that the non-Hermitian degree is introduced in the *σ*_*x*_-component. So, the ${\mathscr{P}}{\mathscr{T}}$ operator is given by ${\mathscr{P}}{\mathscr{T}}={\sigma }_{z}K$, where parity operator inverts all components of quasi-momentum. Depending on the specific form of *d*_*x*_, the system may have ${\mathscr{P}}$ and ${\mathscr{T}}$ symmetries separately. Fortunately, the system has always joint ${\mathscr{P}}{\mathscr{T}}$ symmetry independent of the specific form of *d*_*x*_. The corresponding energy eigenvalues for this Hamiltonian are given by ${E}_{\mp }=\mp \,\sqrt{{(\lambda \mp \cos {k}_{y})}^{2}+{\sin }^{2}\,{k}_{x}-{g}^{2}{d}_{x}^{2}}$. In the Hermitian limit, Dirac points occur if |*λ*| < 1 while the system is gapped if |*λ*| > 1. Suppose here |*λ*| < 1. Changing *λ* shifts the positions of Dirac points. In the non-Hermitian case, the band pattern are changed but the Dirac points are still present. If *d*_*x*_ = 1, then complex energy eigenvalues always appear around the Dirac points. If *d*_*x*_ = sin *k*_*x*_, then we get fully real spectrum if *g* < 1. These Dirac points in non-Hermitian system are topologically robust as long as the ${\mathscr{P}}{\mathscr{T}}$ symmetry is preserved even if the individual ${\mathscr{P}}$ and ${\mathscr{T}}$ symmetries are broken. If the ${\mathscr{P}}{\mathscr{T}}$ symmetry breaking perturbation is added to the system, then the energy gap is opened and the topological Dirac points disappear.

So far, we have studied topological phase for parity-time symmetric systems. Let us briefly mention ${\mathscr{C}}{\mathscr{P}}$ symmetric systems. Consider the following two-dimensional tight-binding non-Hermitian Hamiltonian on the square lattice: ${\mathcal H} (k)=sin\,{k}_{x}\,{\sigma }_{x}+sin\,{k}_{y}\,{\sigma }_{y}+ig\,{d}_{z}({k}_{x},{k}_{y})\,{\sigma }_{z}$, which describes a nodal superconductor with point nodes at the four time-reversal invariant momenta in the Hermitian limit. According to the table given in^29^, this systems has *Z*_2_ topological phase in the limit *g* = 0. Firstly, we note that this Hamiltonian is ${\mathscr{P}}{\mathscr{T}}$ symmetric: ${\mathscr{P}}{\mathscr{T}}={\sigma }_{x}K$. Note that parity sends (*k*_*x*_, *k*_*y*_) → (−*k*_*x*_, −*k*_*y*_). As mentioned before, there is another definition of parity symmetry, where (*k*_*x*_, *k*_*y*_) → (−*k*_*x*_, *k*_*y*_). In this case, the ${\mathscr{P}}{\mathscr{T}}$ operator for the above Hamiltonian becomes ${\mathscr{P}}{\mathscr{T}}=K$ if *d*_*z*_(*k*_*x*_, −*k*_*y*_) = −*d*_*z*_(*k*_*x*_, *k*_*y*_). With this choice of the parity operator, the above Hamiltonian has ${\mathscr{C}}{\mathscr{P}}$ symmetry if *d*_*z*_(*k*_*x*_, −*k*_*y*_) = *d*_*z*_(*k*_*x*_, *k*_*y*_), where ${\mathscr{C}}{\mathscr{P}}={\sigma }_{z}K$. Therefore, the Hamiltonian has either ${\mathscr{P}}{\mathscr{T}}$ or ${\mathscr{C}}{\mathscr{P}}$ symmetries depending on odd or even character of *d*_*z*_. The following two choices satisfy the condition for the ${\mathscr{C}}{\mathscr{P}}$ symmetry: *d*_*z*_ = 1 and *d*_*z*_ = sin *k*_*x*_. The Dirac points of the Hermitian limit are not stable for the former choice since they have complex energy. The Dirac points shift to other positions in the presence of gain and loss. These are exceptional Dirac points and the spectrum is complex around these points. For the latter choice *d*_*z*_ = sin *k*_*x*_, the spectrum is real as long as *g* < 1 and the Dirac points are stable and protected by the parity-time symmetry. At *g* = 1, the Dirac points are turned into three Dirac lines since band touching occurs at *k*_*y*_ = 0, $\mp$*π* and at every points of *k*_*x*_.

To sum up, we have introduced ${\mathscr{P}}{\mathscr{T}}$ symmetry protected topological phase in non-Hermitian systems. We have shown that topological phase exist in non-Hermitian ${\mathscr{P}}{\mathscr{T}}$ symmetric systems. We have given some examples in one and two dimensional systems. In 1*D*, we have shown that stable topological phase exists for topologically insulating system. In 2*D* we have considered gapless systems and found stable Dirac points protected by ${\mathscr{P}}{\mathscr{T}}$ symmetry.
